# The Use of Protected Fat Supplement on Milk Production, Quality and Fatty Acid Profiles in Dairy Camels: Protected Fat Supplement of Dairy Camel

**DOI:** 10.1002/vms3.70351

**Published:** 2025-04-22

**Authors:** Tahereh Mohammadabadi, Morteza Chaji, Somayeh Hoseini, Siamak Amin Davar

**Affiliations:** ^1^ Department of Animal Science Faculty of Animal Science and Food Technology, Agricultural Sciences and Natural Resources University of Khuzestan, Mollasani Ahvaz Iran

**Keywords:** dairy camel, fatty acid profile, milk quality, protected fat

## Abstract

This experiment was conducted to investigate the effect of the protected fat supplement of unsaturated fatty acids in dairy camels. Twenty‐eight Arab camels were fed with control diet and diets containing linolenic acid (omega‐3). The obtained results were analysed as a completely randomized design. Milk production, fat, protein and the antioxidant activity of raw milk increased by using protected fat, but ash, solids and density decreased (*p* < 0.05). Fat, protein, density and the antioxidant activity of fermented milk of camels increased, but solids and lactose decreased (*p* < 0.05). The total microbial load and population of moulds in raw and fermented milk of camels fed with protected fat decreased (*p* < 0.05), but the population of *Lactobacillus* increased (*p* < 0.05). The concentration of unsaturated fatty acids C18:1, C18:2 and C18:3 and conjugated linoleic acid (CLA) increased (*p* < 0.05). Therefore, it may be possible to use omega‐3 protected fat supplementation of 80 g per day in dairy camels feeding in the desert.

## Graphical abstract

Omega‐3 protected fat. Dairy camels were fed with control diet and diets containing 80 g linolenic acid‐protected (omega‐3) supplement for 1 month. The inclusion of 80 g of omega‐3 protected fat in the daily diet of dairy camels in the desert led to an increase in milk production, as well as improved milk fat and protein content. This dietary adjustment also enhanced antioxidant activity and reduced microbial load and mould presence in the milk. Additionally, it raised the concentration of unsaturated fatty acids and conjugated linoleic acid (CLA).

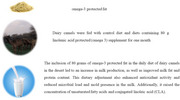

## 1 Introduction

Adding fat to the diet of ruminants may increase the energy density of the diet without increasing the use of cereals as an energy source. Fats have a higher energy value than other nutrients, which are the most important source of energy storage for animals (Sousa et al. 2020). The use of fatty acid calcium salts in addition to providing the energy needed for milk production delivers unsaturated fatty acids (Behan et al. 2019).

Protected fat or calcium salts of fatty acids include amounts of saturated fatty acids such as palmitic and stearic and unsaturated fatty acids such as oleic, linoleic and linolenic (dos Santos Neto et al. 2021). Considering that linoleic acid constitutes 2%–6% of the total fatty acids of milk, in order to provide the required amount, the feed should contain high amounts of protected unsaturated fatty acids (Djordjevic et al. 2019; Giesy et al. 2002).

Protected or calcium salts of fatty acids are made from soy oil, fish oil, flax oil or palm oil, in such a way that triacylglycerols are reacted with calcium compounds. Protected fat shows resistance at pH above 6.5 and protects fatty acids from gastric fermentation. These compounds improve the digestibility of fibre in fat‐containing diets by forming insoluble soaps and protecting fatty acids from decomposition in the stomach (Behan et al. 2019).

In dairy cows, the use of a fat capsule containing approximately 45% palmitic acid, 45% stearic acid and 10% oleic acid has been shown to increase milk production and maintain fat % (Giesy et al. 2002). The use of unsaturated fatty acids in diet leads to a decrease in short‐ and medium‐chain fatty acids in milk but increases long‐chain fatty acids. The conjugated linoleic acid (CLA) as long‐chain fatty acids has positive therapeutic properties in humans (Vargas‐Bello‐Pérez et al. 2019; Pariza et al. 2001).

Using linolenic acid reduced C18:3 fatty acid content by 72.2%, whereas it was not effective on C18:1 *cis*‐9 (Bai et al. 2017). The use of high and low levels of unsaturated fatty acids (stearic acid or protected oleic acid) in the early lactation of dairy cows had different effects on milk reproduction and milk fat production, and stearic acid was more effective for improving milk production (Chen et al. 2019).

Incorporation of calcium palm salts increases total fat digestibility and improves milk yield and milk fat by 3.5% in early and late lactation in dairy cows (dos Santos Neto et al. 2021). The use of fat calcium salts in the ewe did not change fat and SFA, and short‐chain fatty acids increased significantly, and PUFA and MUFA levels decreased (Lurueña‐Martínez et al. 2009). 18‐c unsaturated fatty acids may lead to the prevention of cardiovascular diseases and other chronic diseases (Jiang et al. 2022; Pariza et al. 2001).

Supplementing protected fat increases energy consumption, production and unsaturated fatty acids in milk and finally can have a significant impact on the health of consumers of dairy products (Invernizzi et al. 2016). Considering that the information on the use of protected fat in milk of camels is limited, therefore, this experiment will be conducted to evaluate the effectiveness of protected fat unsaturated fatty acids in nourishing milk of camels.

## 2 Materials and Methods

### 2.1 Animals and Diets

Twenty‐eight dairy camels in fourth lactation, 450 kg body weight (BW), and 1.5 L milk yield were used in a completely randomized design with 2 treatments and 14 replicates. The study lasted 45 days, consisting of a 14‐day adaptation period followed by 30 days of treatment and data collection. The camels were in second and third months of lactation and were healthy.

In this experiment, camels were fed with two control diets and a diet containing 80 g of calcium salt or coated supplement of omega‐3 (19% linolenic acid, EPA, DHA) for 1 month. Protected fat amount counted according to dry matter intake and division by the company.

Camels were fed with pasture forage plants including mesquite, legji, kharsheter and flos before starting the experiment. About 250 g of barley flour, 250 g of wheat flour, 1.5 kg of barley seeds and 3 kg of wheat bran were given to camels daily. The animals had admittance daily to consumption of water and salt licks during the experiment. The basal diet grazed by dromedary camels is shown in Table 1.

**TABLE 1:** Experimental diets components (kg).

Items	kg
Wheat bran	3
Barley	1.5
Barley flour	250 g
Wheat flour	250 g
Prosopis	Free grazing
Camelthorn	Free grazing
Caper	Free grazing
*Cassia*	Free grazing
Calcium carbonate	0.2
Salt	0.3
Vitamin and mineral supplement	0.5

### 2.2 Sample Collection

Milk production was recorded at every milking, twice a day, morning and evening. At the end of the week, sampling was done from the mixed milk of each animal to measure the composition of the milk. The fat, protein, lactose, ash and water of milk were measured by milk analyser device, Milkoscan S50 device (Khalaji et al. 2007).

Fat, protein and lactose were assessed by infrared spectrophotometry (Foss Electric, Hillerod, Denmark). The fatty acid parameters of removed lipids were assessed by gas chromatography as earlier described by Cruz‐Hernandez et al. (2007).

The technique of Çam et al. (2009) was used to assess antioxidant activity through 2,2‐diphenyl‐1‐picrylhydrazyl (DPPH) searching system. First, DPPH methanolic solution with a concentration of 0.06 mM was prepared (due to the sensitivity of DPPH solution to light, the balloon was covered with aluminium foil), and it was made up to volume with methanol and mixed well. Then 1 mL of each sample was poured into the test tube, and 3 mL of DPPH solution was added to it, and immediately the lid of the tubes was covered with foil and mixed well, and after being placed in the dark for 30 min (in order to achieve stable absorption), absorbance of the sample was read at a wavelength of 515 nm. The measurement of the antioxidant property of the samples was calculated using DPPH free radicals according to the following equation.

Percentage of DPPH scavenging activity was calculated as follows: DPPH scavenging activity (%) = [(A blank − A sample)/A blank] × 100, where A is the absorbance.

To investigate the microbial load of raw and fermented milk, 9 mL of physiological serum was poured into seven tubes and autoclaved in order to prepare the appropriate culture dilution. After chilling, sequential dilutions up to 10−7 for wholly treatments were organized. To govern the total count and *Lactobacillus* spp., 1 mL of respective dilution was engaged separately and cultured in a plate (1 mL with a sterile pipette poured into a sterile container of the culture medium of Kant agar and MRS agar plate, which is close to coagulation at a temperature of about 40°C–50°C) and incubated at 31°C for 3 days for total count and 37°C for 48 h for *Lactobacillus* spp. The quantity of colonies was calculated, the totalled average was calculated in diverse dilutions, and the whole count was measured with *Lactobacillus* spp. of milk.

The number of moulds through the surface culture in PDA medium at 25°C for 48 h, measurement and results reported to the number of colonies obtained from each millilitre of milk (colony count/mL). The number of colonies is counted, and the average counted in different dilutions is calculated. The yeast mould population of raw and fermented milk was measured, and the results were reported as the number of colonies per millilitre through appropriate dilution surface culture in PDA culture medium at 25°C for 48 h (Khalaji et al. 2007).

### 2.3 Preparation of Fermented Milk

In order to ferment camel milk, the milk was kept in anaerobic conditions at 40°C for 48 h so that the milk fermentation is done naturally, and then after passing the desired time for fermentation, microbial tests, antioxidant activity and fermented milk compounds were measured (Sotoudeh et al. 2018).

### 2.4 Fatty Acid Profile

Fatty acid profile was assessed by gas chromatography. After derivatization, fatty acid methylated esters were produced, and fatty acids were determined by gas chromatography. A Varian 3400 gas chromatograph (Ajax, Canada) equipped with an FFAP column of 100‐m length and 0.25 µm thickness at a column temperature of 215°C or 225°C as described by Cruz‐Hernandez et al. (2007).

This test, which is according to the method of the national standards of Iran, is numbered 13126‐2 and 13126‐4. Two cm^3^ milk is taken from each milk sample and mixed with 2 cm^3^ of hexane, and then the milk fat is added to the hexane and then takes 1 cm^3^ of hexane and adds 200 µmol of 2 M methanolic potash to it and vortex and wait for 5 min until the two phases are completely separated. We inject the obtained top into the GCFID device to report the amount and type of fatty acids separately.

### 2.5 Statistical Analysis

The results were analysed as a completely randomized design (*t*‐test) using the General Linear Model (GLM) procedure of the SAS software (version 9.2). Means were compared by the Tukey multiple comparison tests at *p* < 0.05. The model of this design is a completely randomized design with two treatments, and each treatment includes five replications based on the statistical model: *Y_ij_* *= μ + T_i_* *+ e_ij_*. Where *Y_ij_* is the observation, *μ* is the general mean, *T_i_* is the effect of treatments, and *e_ij_* is the standard error of the term.

Moreover, repeated data per time were analysed as repeated measurements using the MIXED procedures of SAS based on the statistical model: *Y_ijk_ = μ + T_i_* *+ H_j_* *+ (T × H)_ij_* *+ e_ijk_*. Where *Y_ijk_* is the observation, *μ* is the mean of observations, *T_i_* is the effect of treatments, *H_j_* is the effect of sampling day, *(T* × *H)_ij_* is interactions between the effect of treatments and sampling day, and *e_ij_* is the standard error of term.

## 3 Results

### 3.1 Milk Production and Quality

The results of production and compositions of milk are given in Table 2. Milk production, fat and protein increased, whereas ash, solid content and density decreased (*p* < 0.05). Lactose was not affected by the experimental treatment (*p* > 0.5).

**TABLE 2:** Effect of protected fat supplementation on milk production and composition of Arabian camels (%).

Composition	Control	Protected fat	SEM	*p* value
Milk production (L)	2.7^b^	4.3^a^	0.22	0.05
Fat	2.40^b^	4.27^a^	0.22	0.02
Density	31.78^a^	26.50^b^	0.31	0.03
Ash	0.62^a^	0.57^b^	0.01	0.017
Fat‐free solid	8.18^a^	7.55^b^	0.01	0.03
Lactose	4.54	4.36	0.24	0.65
Protein (%)	2.79^b^	3.13^a^	0.015	0.003
Freezing point	0.54^a^	0.49^b^	0.003	0.012

*Note*: Different letters in each row indicate a significant difference (*p* < 0.05).

The composition of the fermented milk of camels is given in Table 3. Fat, protein and density of fermented milk increased and the content of non‐fat solids and lactose decreased (*p* < 0.05), but the ash in fermented milk was not influenced (*p* > 0.05).

**TABLE 3:** The effect of the treatments on the composition of fermented camel milk (%).

Composition of milk	Control	Fat	SEM	*p* value
Fat	1.62^b^	3.11^a^	0.06	0.0041
Density	25.72^b^	34.25^a^	0.35	0.0081
Ash	0.79	0.83	0.15	0.21
Fat‐free solids	8.12	8.79	0.17	0.11
Lactose	3.26^a^	3.11^b^	0.01	0.04
Protein (%)	3.5^b^	3.94^a^	0.16	0.02
Freezing point	0.31^a^	0.28^b^	0.19	0.18

*Note*: Different letters in each row indicate a significant difference (*p* < 0.05).

The results of antioxidant properties using DPPH scavenging activity in raw and fermented milk in camels fed with experimental diets are given in Table 4. The antioxidant activity of raw and fermented milk was higher than that of control (*p* < 0.05).

**TABLE 4:** The effect of the examined treatments on the antioxidant property (DPPH) of raw milk and fermented camel milk (%).

	Control	Fat	SEM	*p* value
Raw milk	48.99^a^	40.25^b^	0.55	<0.0001
Fermented milk	25.58^a^	20.54^b^	0.66	<0.0001

*Note*: Different letters in each row indicate a significant difference (*p* < 0.05).

According to the results, total microbial load in raw (Table 5) and fermented (Table 6) milk of camels fed fat decreased (*p* < 0.05). Accumulation of mould in raw and fermented milk in camels fed fat decreased compared to control, but the amount of *Lactobacillus* spp. increased (*p* < 0.05).

**TABLE 5:** The effect of the investigated treatments on the microbial population of camel milk (Log CFU/mL).

Microbial population	Control	Fat	SEM	*p* value
Total microbial load	4.38^a^	3.99^b^	0.09	0.02
Yeast mould	3.41^a^	2.92^b^	0.12	0.03
*Lactobacillus*	3.48^b^	4.68^a^	0.5	<0.0001

*Note*: Different letters in each row indicate a significant difference (*p* < 0.05).

**TABLE 6:** The effect of the examined treatments on the microbial population of fermented camel milk (Log CFU/mL).

Microbial population	Control	Fat	SEM	*p* value
Total microbial load	8.39^a^	7.34^b^	0.11	0.0005
Yeast mould	8.24^a^	7.63^b^	0.03	0.002
*Lactobacillus*	8.08^b^	8.48^a^	0.06	0.007

*Note*: Different letters in each row indicate a significant difference (*p* < 0.05).

### 3.2 Milk Fatty Acids

The fatty acid profile of milk in the camels fed protected fat is given in Table 7. The results showed that the use of protected fat increased the concentration of short‐chain fatty acids C:8, C:10, C:6, C:4 and decreased the medium‐chain fatty acids C:14, C:12 (*p* > 0.05). Using protected fat increased the concentration of unsaturated fatty acids C18:1, C18:2, C18:3 and decreased saturated fatty acids C:18 and C:16 in milk compared to the treatment (*p* > 0.05). Moreover, according to the results, the use of protected fat had a significant effect on the increase in CLA levels (*p* < 0.05).

**TABLE 7:** The effect of the investigated treatments on the fatty acid profile of camel milk (%).

Fatty acid	Control	Bat	SEM	*p* value
C2:0	0	14.5	0.35	0.0012
C: 4	61.55^b^	64.7^a^	0.11	0.0043
C: 6	1.15^b^	1.55^a^	0.05	0.02
C: 8	3.05^b^	5.35^a^	0.05	0.0009
C: 10	2.3^b^	3.6^a^	0.01	0.011
C: 12	2.45^a^	1.4^a^	0.21	0.02
C: 14	1.25^a^	0.85^b^	0.05	0.029
C16:0	4.1^a^	3.3^b^	0.1	0.02
C16:1	0.52	0.51	0.30	0.21
C18:0	1.45^a^	1.05^b^	0.11	0.03
C18:1	1.45^a^	2.75^a^	0.05	0.002
C18:2	0.15^a^	0.35^b^	0.05	0.1
C18:3	0.29^b^	0.59^a^	0.01	0.002
C20	0.54	0.49	0.41	0.94
CLA	0.48^b^	1.3^a^	0.07	0.01

*Note*: Different letters in each row indicate a significant difference (*p* < 0.05).

## 4 Discussion

According to the current study, using protected fat increased milk production, fat and protein. The effect of fat supplements on the amount of milk production depends on many factors such as the basic ration, stage of lactation, energy balance, fat composition and the amount of fat supplement (NRC 2001). In most of the studies on dairy cows, by adding different sources of fat to the diet, the amount of energy in the diet increases, and milk production is increased.

Nasiri et al. (1400) reported that the use of 390–1000 g of protected fat improved energy conversion efficiency in lactating cows and increased milk production. Protected fat supplements increase milk production by reducing glucose consumption. They do this by preventing the daily synthesis of fatty acids in the mammary gland, possibly stopping glucose oxidation and using excess glucose for lactose synthesis.

Protected fats reduce probable interference with rumen microbial fermentation; hence, complete fat breakdown occurs only in the udder (Ibrahim et al. 2021). Feeding protected fats increases the availability of fatty acids in the intestine and mammary gland for milk fat synthesis. Protected fats increase fibre digestion and dry matter intake and improve milk production and milk fat content of dairy cows (Ibrahim et al. 2021).

According to studies, supplementation of 4%–6% of protected fats increased production and milk fat in high‐producing dairy cows (Kundu et al. 2014). The different response of animals to protected fat is due to the difference in palatability, the level entered in the diet, and their solubility and melting point in the rumen (Purushothaman et al. 2008).

In dairy cows, the use of encapsulated fat containing approximately 45% palmitic acid, 45% stearic acid and 10% oleic acid (sources of unsaturated fatty acids) increased milk production and maintained milk fat percentage (Giesy et al. 2002). Titi (2011) observed an increase in the percentage of milk compounds and milk production by using 3% and 5% calcium salts or protected fat from early to mid‐lactation in Shami goats. Ranaweera et al. (2019) reported that the effect of protected fat supplementation on milk production of tropical dairy cows from the time of birth to the 15th week of lactation was higher with a dosage of 200 g per cow per day, but the amount of milk fat, fat‐free solids and protein was not affected by the protected fat supplement.

In investigating the effect of supplementing protected fat at three levels of 250, 350 and 450 g per day in dairy cows and buffaloes, milk production and milk fat increased at levels of 250 and 350. In a study on native Pakistani Sahiwal cows, supplementing protected fat can significantly increase milk and fat production (Mobeen et al. 2016). Ground flaxseed containing protected fat could increase milk production. Linolenic acid released from flaxseed can increase gluconeogenesis, and as a result, milk lactose concentration and the milk production increase (Mashek and Grummer 2003).

Ghahramani et al. (2020) reported that the addition of protected fat sources and calcium fatty acids increases the percentage of milk fat. The milk fat produced in cows fed with protected fat supplement was higher than the control group between the 4th and 10th months of the experiment. Moreover, the milk protein and lactose were higher in the whole period in cows fed with protected fat supplement than in the control treatment (Jolazadeh et al. 2019a).

According to the research, the feeding of extruded flaxseed with protected fat from birth to 40 days after birth compared to calcified palm oil had no effect on the production of milk, protein and milk lactose, but it reduced the percentage of milk fat, which may be due to the higher consumption of long‐chain unsaturated fatty acids (Dirandeh et al. 2013). In the study of Moalem et al. (1999), milk production was higher in the treatments that consumed protected fat supplements (saturated and unsaturated), and the percentage of milk protein was lower compared to the control.

Feeding oilseeds or protected fat decreases the milk protein concentration. Although the mechanisms are not yet clear, one reason could be the reduction of dietary glucogenic precursors that have been replaced by fat (Ganj Khanlou et al. 2015). Most studies have reported a lack of response to lactose when using fat supplements. Due to the fact that lactose regulates the osmotic pressure in the mammary gland and water diffuses into the breast tissue, the concentration of lactose remains constant up to 95%. But supplementation of protected fat led to a decrease in milk fat (Sultana et al. 2008).

In the protected fat group, antioxidant activity of milk was higher than that of control. Mitsiopoulou et al. (2021) investigated the effect of whole sesame seeds with protected fat at two levels of 5% and 10% of the diet on the antioxidant status of goat milk. Catalase and superoxide dismutase activity increased in the blood of goats supplemented with 10% whole sesame seeds. The obtained results state that the inclusion of high levels of whole sesame seeds in the diet of goats can improve the oxidative stability of milk and the antioxidant status.

Protected fat supplementation significantly increased alpha‐tocopherol by 56% after 21 days, which has antioxidant properties. Vitamin E and alpha‐tocopherol can prevent the production and increase of malondialdehyde in milk. Addition of linseed to the diet led to an increase of alpha‐tocopherol in milk, which is due to its high transfer from seeds to milk, which can increase the oxidative stability of milk. These supplements can increase antioxidant compounds, including enzymes in milk in a short period of time, which can reduce oxidation in milk (Pappel et al. 2013).

According to studies, it has been reported that the antioxidant properties of milk are due to the presence of antioxidant enzymes, glutathione peroxidase, superoxide dismutase, as well as lactoferrin, lysozyme, casein, lactoperoxidase and beta‐lactoglobulin and two isomers of CLA and butyric acid, which can be directly transferred from feed to milk (Pappel et al. 2013). Atashak et al. (2012) reported that different parts of whey in camel milk fermented with *Lactobacillus rhamnosus* had higher antioxidant activity than cow's milk. By supplementing protected fat, the amount of flavonoids in milk increases.

Total microbial load and mould in camels’ milk fed protected fat decreased, but *Lactobacillus* spp. increased. According to studies, during yogurt fermentation, the population of lactobacilli increased. But adding the protected fat supplement could not affect the population of lactobacilli, and the number of moulds remained unchanged (Kalyas and Ürkek 2022). Ganj Khanlou et al. (2015) reported that the population of coli forms and mould and yeast in milk enriched with protected fat supplements did not change.

Feeding protected fat increased the concentration of short‐chain fatty acids, unsaturated fatty acids and CLA levels and decreased saturated fatty acids in camel milk compared to the control. Using sources rich in omega‐3 and omega‐6 did not change the percentage of short‐ and medium‐chain fatty acids and C18:1 trans fatty acids. However, it was able to change C18:1 and C18:2 isomers individually in milk fat (Glasser et al. 2008). Feeding extruded soybeans as a source of linoleic acid and protected fat or calcium salt of long‐chain fatty acids did not affect the percentage and production of C4:0, C6:0 and C16:1 and the production of C8:0, C10:0, C12:0, C14:1, C15:0 and C17:0 decreased and the percentage of C18:0, C18:1, C18:2 and C18:3 increased (Schauff et al. 1992).

The research showed that the feeding of rolled soybeans with protected fat during different periods before birth caused an increase in *cis*‐9, *cis*‐12, *cis*‐9, *trans*‐11 and total fatty acids with several double bonds, but there was no significant effect on short‐ and medium‐chain fatty acids (Gardinal et al. 2016). Feeding the protected fat supplements increased the proportion of fatty acids with double bonds and decreased saturated fatty acids in milk fat. In terms of human health, these changes improve the fatty acid profile of milk.

Some researchers attributed the change in the pattern of milk fatty acids to the negative effects of fat on cellulolytic bacteria. Because many of the medium‐chain fatty acids are synthesized de novo in the mammary gland from the acetate obtained from rumen metabolism, in addition, mono carbonated fatty acids are produced by rumen bacteria and secreted directly into the milk.

An increase in the amount of CLA in the meat of lambs fed diets containing 5% safflower seeds containing protected fat was reported by Bolte et al. (2002). Therefore, feeding dairy cows with whole flax seeds, soybean oil, or soybean meal represents a successful strategy to improve the fatty acid composition of yogurt by increasing omega‐3 fatty acids and reducing the ratio of omega‐6 to omega‐3 in milk and yogurt (Amer et al. 2018).

In cows fed linolenic acid supplement, the content of linoleic fatty acid decreased by 2.8% and omega‐3 fatty acids of milk decreased by 2.72% (Bai et al. 2017). The use of protected fat or calcium salts of omega‐3 and omega‐6 fatty acids in the last 3 weeks of pregnancy in Holstein cows led to an increase in the amount of omega‐3 and omega‐6 in colostrum (Jolazadeh et al. 2019b).

Supplementing the protected fat or calcium salt of fish oil increased the total *n*‐3 PUFA fatty acids (including C18:3 *n*‐3, C20:5 and C22:6) in meat, and this caused a decrease in the ratio of *n*‐6/*n*‐3 fatty acids and increase omega‐3 fatty acids (eicosapentaenoic acid and docosahexaenoic acid) were found in the muscle, which has a significant effect on increasing the nutritional value of meat. The use of flax seeds in the diet can increase the amount of omega‐3 in milk. Flaxseed reduces palmitic acid and increases CLA (Petit 2015).

Nasiri et al. (1400) reported that the amount of milk linoleic acid in the diet containing protected fat supplement was higher than the control diet. Feeding a protected fat supplement decreased short‐ and medium‐chain fatty acids and increased long‐chain fatty acids. In the study of Kim et al. (1993), feeding protected fat or calcium salts of fatty acid and extruded soy increased the amount of unsaturated fatty acids in milk, which was attributed to the escape of fatty acids from rumen biohydrogenation.

It is proved that long‐chain fatty acids of milk reduce the activity of acetyl‐CoA carboxylase enzymes or by preventing short or medium‐chain fatty acids from attaching to the two and three positions of glycerol. The protected fat supplement slightly affected rumen biohydrogenation and probably reduced the daily synthesis of fatty acids in the mammary gland through the inhibition of acetyl coenzyme A carboxylase. In the mammary gland of ruminants, monounsaturated fatty acids are increased through direct absorption or by the unsaturation of saturated fatty acids by the enzyme delta 9 desaturase (Benson et al., 2001). Alperen and Bayram (2022) reported that after supplementing the diet with linseed or rapeseed containing protected fat, a decrease in saturated fatty acids and an increase in omega‐3 and omega‐6 levels were observed.

## 5 Conclusion

According to the results of a recent study, the use of omega‐3 protected fat supplement in the diet of dairy camels increased milk production, milk fat and protein, antioxidant activity and reduced microbial load and moulds in milk. Moreover, the concentration of unsaturated fatty acids and CLA increased. Therefore, it may be possible to use omega‐3 protected fat supplement as a suitable supplement at the rate of 80 g per day in the feeding of dairy camels in the desert.

## Author Contributions

**Tahereh Mohammadabadi and Morteza Chaji**: conceptualization. **Tahereh Mohammadabadi**: data curation. **Tahereh Mohammadabadi and Somayeh Hoseini**: formal analysis. **Tahereh Mohammadabadi, Somayeh Hoseini, Siamak Amin Davar and Morteza Chaji**: funding acquisition. **Tahereh Mohammadabadi, Somayeh Hoseini, Siamak Amin Davar and Morteza Chaji**: investigation. **Tahereh Mohammadabadi and Morteza Chaji**: methodology. **Tahereh Mohammadabadi**: project administration. **Tahereh Mohammadabadi, Morteza Chaji and Siamak Amin Davar**: resources. **Tahereh Mohammadabadi**: software. **Tahereh Mohammadabadi**: supervision. **Tahereh Mohammadabadi and Morteza Chaji**: validation. **Tahereh Mohammadabadi and Morteza Chaji**: visualization and writing–original draft and Writing–review and editing.

## Disclosure

The manuscript did not involve human subjects or human transplantation studies, and no organs/tissues were obtained from the prisoners. This manuscript did not contain any individual person**’**s data in any form (including any individual details, images or videos).

## Ethics Statement

All measures and procedures concerning animals were permitted by the Animal Experiment Committee of Agricultural Sciences and Natural Resources University of Khuzestan, Mollasani, Iran. (Approval no. ASD 008/1403). All animal management and sampling procedures conducted according to The Care and Use of Agricultural Animals in Research and Teaching guidelines (FASS 2010). The authors confirm that the ethical policies of the journal, as noted on the journal's author guidelines page, have been adhered to and the appropriate ethical review committee approval has been received. The authors confirm that they have followed EU standards for the protection of animals used for scientific purposes [and feed legislation, if appropriate].

## Conflicts of Interest

The authors declare no conflicts of interest.

### Peer Review

The peer review history for this article is available at https://www.webofscience.com/api/gateway/wos/peer‐review/10.1002/vms3.703514.

## Data Availability

The data that support the findings of this study are available from the corresponding author upon reasonable request.
